# The Contrast Enhancement of Intracranial Arterial Wall on High-resolution MRI and Its Clinical Relevance in Patients with Moyamoya Vasculopathy

**DOI:** 10.1038/srep44264

**Published:** 2017-03-09

**Authors:** Maoxue Wang, Yongbo Yang, Fei Zhou, Ming Li, Renyuan Liu, Maobin Guan, Rui Li, Le He, Yun Xu, Bing Zhang, Bin Zhu, Xihai Zhao

**Affiliations:** 1 Department of Radiology, The Affiliated Drum Tower Hospital of Nanjing University Medical School, Nanjing 210008, China; 2 Department of Radiology, Nanjing Xianlin Drum Tower Hospital, Nanjing 210046, China; 3 Department of Neurosurgery, The Affiliated Drum Tower Hospital of Nanjing University Medical School, Nanjing 210008, China; 4 Department of Radiology, Yangzhou First People’s Hospital, Yangzhou 225001, China; 5 Center for Biomedical Imaging research, Department of Biomedical Engineering, Tsinghua University School of Medicine, Beijing 100084, China; 6 Department of Neurology, The Affiliated Drum Tower Hospital of Nanjing University Medical School, Nanjing 210008, China

## Abstract

The purpose of this study is to investigate the characteristics of intracranial vessel wall enhancement and its relationship with ischemic infarction in patients with Moyamoya vasculopathy (MMV). Forty-seven patients with MMV confirmed by angiography were enrolled in this study. The vessel wall enhancement of the distal internal carotid artery, anterior cerebral artery and middle cerebral artery was classified into eccentric and concentric patterns, as well as divided into three grades: grade 0, grade 1 and grade 2. The relationship between ischemic infarction and vessel wall enhancement was also determined. Fifty-six enhanced lesions were found in patients with (n = 25) and without acute infarction (n = 22). The incidence of lesions with grade 2 enhancement in patients with acute infarction was greater than that in those without acute infarction (*p* = 0.011). In addition, grade 2 enhancement of the intracranial vessel wall was significantly associated with acute ischemic infarction (Odds ratio, 26.7; 95% confidence interval: 2.8–258.2; *p* = 0.005). Higher-grade enhancement of the intracranial vessel wall is independently associated with acute ischemic infarction in patients with MMV. The characteristics of intracranial vessel wall enhancement may serve as a marker of its stability and provide important insight into ischemic stroke risk factors.

Moyamoya vasculopathy (MMV) is characterized by progressive stenosis of the intracranial internal carotid artery (ICA) and its proximal branches^1^. MMV can be manifested as Moyamoya disease (MMD), unilateral MMD and quasi-MMD. MMD is usually an idiopathic and bilateral disease. Unilateral MMD is defined as MMD with unilaterally affected vessels and not associated with any other underlying conditions. Bilateral or unilateral MMD angiographic findings associated with a definite underlying disease, such as atherosclerosis, is usually categorized as quasi-MMD^2^,^3^. The prevalence of MMD, unilateral MMD and quasi-MMD is 5.22/100, 000, 0.66/100, 000 and 0.34/100, 000, respectively^3^. Cerebral ischemia and hemorrhage are the major outcomes of MMV^4^,^5^. However, the risk factors of ischemia and hemorrhage are still controversial.

Clinically, the diagnosis of MMV still relies on angiographic techniques, such as digital subtraction angiography (DSA), MR angiography (MRA) and computed tomography angiography. These approaches focus on measuring the stenosis of arterial lumen which has several limitations. First, the stenosis of arterial lumen does not reflect the underlying pathology of the vessel wall. It is well established that various diseases in the vessel wall can lead to luminal stenosis, such as atherosclerosis, vasculitis, dissection and MMV. In addition, for arterial atherosclerotic disease, the measure of luminal narrowing underestimates disease severity when positive remodeling is present, often presenting as large plaque with normal lumen size or a lower grade stenosis^6^,^7^. Therefore, characterization of the vessel wall is necessary to evaluate etiology, disease severity and the stability for intracranial artery diseases. High-resolution magnetic resonance image (HR-MRI) has become a useful tool in assessing atherosclerotic disease in both intra- and extra-cranial carotid arteries^8^,^9^. Recently, investigators utilized HR-MRI to distinguish MMD from atherosclerosis and found that MMV usually shows shrinkage of the middle cerebral artery (MCA) and concentric enhancement of bilateral distal ICAs and the MCA^10^,^11^,^12^. Previous studies have shown that enhancement of the vessel wall may reflect inflammation and neovascularization that is associated with disease stability and worse clinical outcomes^13^,^14^,^15^.

However, little is known about the characteristics and clinical relevance of intracranial vessel wall enhancement in patients with MMV. Thus, we hypothesized that the enhancement of arterial walls in patients with MMV might exist and might be related to clinical outcome, such as cerebral ischemic infarction. This study sought to investigate the characteristics of the enhancement of MMV by three dimensional (3D) HR-MRI and their relationship with ischemic infarction.

## Results

From October 2013 to December 2014, 51 patients with MMV were consecutively recruited in this study. Four patients were excluded because of poor MR image quality caused by severe motion artifact. Therefore, 47 patients (20 male; 48.7 years ±10) were included in the final analysis. Twenty-five patients had acute infarction. The median interval between symptom onset and MR imaging in patients with cerebral infarction was 45 days (interquartile range, 8 days to 3 months). The median interval between diagnosis of MMV by DSA and HR-MR imaging was 5 days (interquartile range, 1 day to 18 months). Sixteen patients came to the hospital because of extremity adynamia, 15 patients with headache, 12 patients with dizziness, 3 patients with aphasia and 1 patient with numbness. The differences of vascular risk factors between patients with and without cerebral infarction were not significant except hypertension (*p* = 0.047). Clinical characteristics and intracranial findings of patients are detailed in Table 1.

### Characteristics of intracranial vessel wall enhancement

Of 47 patients, 12 were found to have unilateral MMV and 35 bilateral MMV. In total, 246 MMV lesions were detected. Of all MMV lesions, according to the degree of contrast enhancement, 190, 22, and 34 lesions were graded as grade 0, grade 1, and grade 2, respectively. Of the 34 grade 2 lesions, 14 lesions did not have acute infarction in the corresponding vascular area, 6 lesions were in patients with acute infarction and 8 lesions were in patients without acute infarction. Of 25 patients with acute infarction, 5 patients had grade 1 enhancement and 5 patients did not have any enhancement of corresponding vessels. The contrast enhancement was found to be most likely involved in distal ICA (27/56, 48.2%), followed by MCA (18/56, 32.1%) and ACA (11/56, 19.6%). Of all contrast enhanced lesions, concentric enhancement (n = 40, 71.4%) was significantly more common than eccentric enhancement (n = 16, 28.6%) (*p* < 0.001). Characteristics of intracranial vessel wall enhancement are summarized in Table 2.

### Association between intracranial vessel wall enhancement and ischemic event

More contrast enhanced MMV lesions were found in patients with cerebral acute infarction compared to those without acute infarction (based on lesion-level: 66.1% vs 33.9%, *p = *0.011; based on patient-level: 63.2% vs 36.8%, *p* < 0.001). Grade 2 enhancement in intracranial vessel wall was associated with ischemic infarction (odds ratio = 26.7; 95% confidence interval: 2.8–258.2; *p = *0.005) when grade 0 was considered as reference. This association was not found between grade 1 enhancement and ischemic infarction (odds ratio = 4.0; 95% confidence interval: 0.4–44.1; *P* = 0.258).

### Reproducibility

The interreader agreement between the two readers for grading MMV enhancement was excellent (weighted k = 0.801). The intra-reader agreement was also excellent (weighted k = 0.825).

## Discussion

This study investigated the characteristics of intracranial vessel wall enhancement and their relationship with acute cerebral infarction in patients with MMV. We found that the vessel wall enhancement of MMV lesions was more likely located in the distal ICA and most of enhanced lesions appeared concentric enhancement. Furthermore, higher grade enhancement of intracranial vessel walls was found to be independently associated with acute ischemic infarction, suggesting that characteristics of intracranial vessel wall enhancement might provide additional insight to stratify the risk of ischemic stroke in patients with MMV.

We found the concentric enhancement of intracranial vessel wall was commonly present in patients with MMV. This finding has been presented in previous studies. Ryoo *et al*. reported that more than 80% concentric enhancement had been found in the symptomatic MMD segment^10^. The vascular endothelial growth factor (VEGF) and VEGF receptor might act as regulatory factors for angiogenesis including the abnormal vascular network^16^. Chmelova *et al*. found that angiogenetic factors in intima and VEGF in the endothelium of moyamoya-affected arteries were higher expressed than normal^17^. This suggests an active angiogenetic process in the enhanced vessel wall in patients with MMV. In the extracranial vessel, inflammatory conditions have been thought to be associated with concentric, circumferential wall thickening and enhancement with pathologic confirmation^18^. A strong inflammatory response in patients with atherosclerotic plaque reflected increased macrophage infiltration and neovascularity^19^. Our results extend those observations to moyamoya-affected vessels and suggest that enhancement of intracranial vessels in patients with MMV may indicate active angiogenetic process and inflammatory response in patients with MMV.

In this study, the enhancement of the moyamoya-affected vessel wall was found to more likely involve the distal ICA, followed by the MCA and the ACA. Stenosis of the ICA had been thought to be the first lesion in patients with MMV, and then progress to involve the ACA, MCA and sometimes to posterior cerebral arteries^20^. In children, stenosis of the distal ICA, proximal MCA and ACA on one side and the remarkable stenosis of distal ICA on the contralateral side was diagnosed as MMD. However, in adult, MMD has to be diagnosed as stenosis involved in both sides of the distal ICA, proximal MCA and ACA^1^, which may also indicate that ICA was the position of first stenosis.

Hypertension was different in patients with and without acute infarction, and most often was located in patients with acute infarction in this study. Hypertension was one of the vascular risk factors to atherosclerosis which is correlated with stroke. Higher-grade enhancement of moyamoya-affected vessel wall was found to be associated with ischemic infarction in patients with MMV after being adjusted for hypertension. Enhancement of the vessel wall may reflect the level of neovascularization in atherosclerotic patients, which was found to be independently correlated with recent cerebral ischemic events^21^,^22^. In addition, strong contrast enhancement was thought to be related to greater neovascularization both of which facilitate the delivery and accumulation of the contrast agent^23^. A recent intracranial study revealed enhanced atherosclerotic plaque might be instable, and the higher-grade enhancement was independent associated with culprit plaques after adjusted for plaque thickness. This relationship can be used to identify lesions responsible for intracranial ischemic events^24^. In this study, enhancement of the vessel wall may reflect a proliferative neovascular process and the activity of the lesion.

In this study, grade 2 enhancement of the intracranial vessel wall was found to be associated with acute ischemic stroke. Of 25 patients with acute infarction, 5 patients had grade 1 enhancement and 5 patients did not have any enhancement of corresponding vessel. These lesions may have a responsible extracranial vessel, although the DSA was normal. Fourteen lesions with grade 2 enhancement did not have any acute infarction in the corresponding vascular area, which may serve as a risk factor of ischemic stroke in the future. A meta-analysis of 1156 previously symptomatic MMD patients concluded that most patients showed improvement after operation. However, 2.7% of patients had definite deterioration^25^. For these 2.7% patients, the vessel wall might be the target treatment before operation to stabilize the vessel wall.

Our study has several limitations. First, pathology of MMD, unilateral MMD and quasi-MMD in our study remains unclear, although it had been reported one patient with quasi-MMD has the same pathological changes with MMD^26^. However, the diagnosis of MMV in this study was based on DSA rather than histology. Second, the exact mechanism accounting for intracranial vessel wall enhancement remains unknown. More studies of molecular contrast agents may be helpful for understanding this mechanism. Third, we only observed the occurrence of cerebral infarction in a cross-sectional study. In the future, prospective studies with large sample sizes are needed to validate the predicting value of intracranial vessel wall enhancement for future ischemic stroke.

In conclusion, the intracranial vessel wall enhancement more likely involves distal ICA and exhibits concentric patterns. The finding of an independent association between strong enhancement of intracranial vessel wall and cerebral infarction suggests that vessel wall enhancement determined by MR vessel wall imaging may provide insight into future stroke risk in patients with MMV.

## Methods

### Study sample

The study protocol was approved by Medical Research Ethics Committee of the Affiliated Drum Tower Hospital of Nanjing University Medical School. The written consent form was obtained from all subjects. Our study was carried out in accordance with the relevant guidelines and regulations. Patients with MMV were recruited in this study. MMV was diagnosed according to the evidence of stenosis in distal ICA, proximal MCA and ACA simultaneously on DSA^27^. The other inclusion criteria included: (1) age from 18 to 80 years old; (2) neurovascular symptoms within 1 year; (3) no revascularization. The exclusion criteria were as follows: (1) potential sources of cardioaortic embolism; (2) ≥50% extracranial stenosis; (3) failure of renal function (glomerular filtration rate <60 mL/min); 4) contraindications to MR imaging. All patients underwent brain imaging and intracranial vessel wall 3D HR-MRI. The clinical information including age, gender, and history of smoking, hypertension, hyperlipidemia and diabetes was collected.

### MR imaging

MRI examinations were performed with a 3.0 Tesla MR scanner (Achieva TX, Philips Medical Systems, The Netherlands) with an 8-channel phased array head coil. The brain MR images were acquired with the following sequences and parameters: diffusion weighted imaging (DWI): b value 1000, time to repetition (TR)/time to echo (TE) 2322/88 ms, flip angle (FA) 90°, field of view (FOV) 230 × 230 mm^2^, matrix size 152 × 121, slice thickness 6 mm and acquisition time 27 s; fluid attenuated inversion recovery (FLAIR): TR/TE, 7000/120 ms, FOV 230 × 230 mm^2^, matrix size 328 × 267, slice thickness 6 mm and acquisition time 1 min 34 s; T1-weighted imaging: TR/TE 250/2.3 ms, FA 75°, FOV 230 × 183 mm^2^, matrix size 256 × 163, slice thickness 6 mm and acquisition time 43 s; T2-weighted imaging: TR/TE 2223/80 ms, FA 90°, FOV 230 × 183 mm^2^, matrix size 328 × 267, slice thickness 6 mm and acquisition time 1 min 33 s; three-dimensional time of flight (3D TOF) MRA: TR/TE 25/3.5 ms, FA 20°, FOV 180 × 180 × 60 mm^3^, matrix size 360 × 360, voxel size 0.47 × 0.47 × 0.5 mm^3^ and acquisition time 4 min 55 s.

The intracranial vessel wall was imaged by pre- and post-contrast T_1_ weighted imaging with sequence of 3D Motion-Sensitized Driven-Equilibrium Prepared Rapid Gradient Echo (MERGE)^28^ using the following parameters: fast field echo, improved Motion-Sensitized Driven-Equilibrium (MSDE)^29^, TR/TE 13/6.6 ms, FA 6°, FOV 160 × 160 × 45 mm^3^, spatial resolution 0.5 × 0.5 × 0.5 mm^3^, and acquisition time 6 min 40 s. Post-contrast 3D MERGE images were obtained 5 minutes later by intravenous administration of Gadodiamide (Gd-DTPA-BMA, Nycomed) with a dose of 0.1 mmol/kg.

### Image review

**Figure 1:**
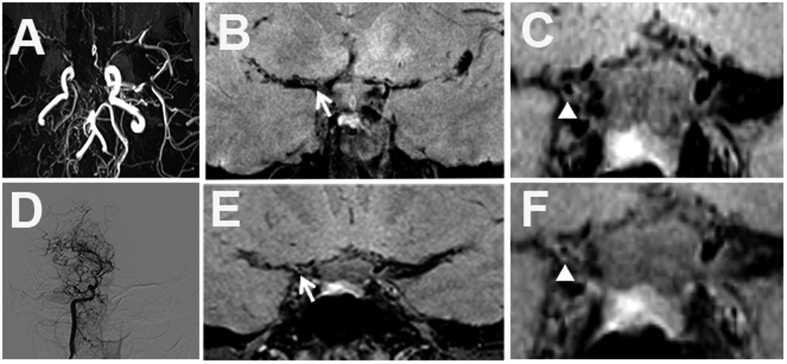
Grade 0 enhancement of MMV. (**A**) Occlusion in right MCA and ACA and stenosis in left MCA on TOF MRA. Stenosis of right distal ICA with no enhancement on pre- (**B,C**) and post-enhanced images (**E,F**). (**D**) Moyamoya vessels in right ICA and branches on DSA.

**Figure 2:**
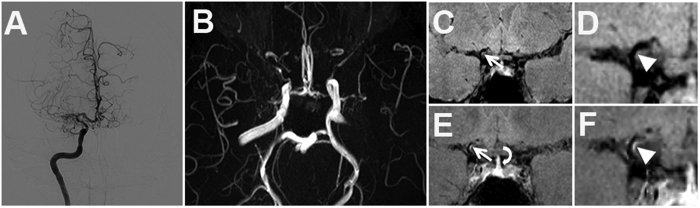
Grade 1 enhancement of MMV. (**A**) Moyamoya vessels in right ICA and branches on DSA. (**B**) Discontinuous MCAs on TOF MRA. (**C,D**) Pre- and (**E,F**) post-enhanced images show the stenosis of right proximal ACA with mild enhancement (white arrow and arrow head) but less than that of pituitary infundibulum (curved arrow).

**Figure 3:**
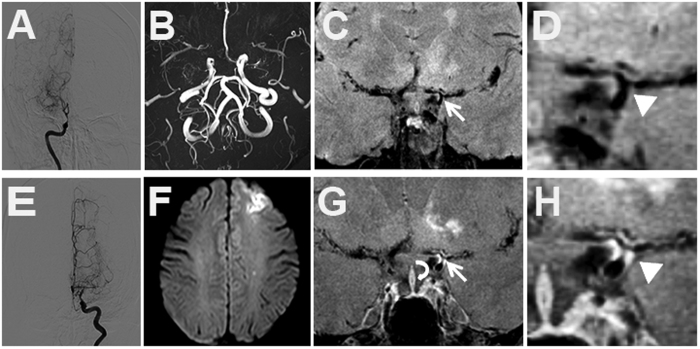
Grade 2 enhancement of MMV. (**A,E**) intracranial vessels on DSA. (**B**) Occlusion of bilateral MCAs on TOF MRA. (**F**) Acute ischemic infarction of left frontal lobe on DWI images. Pre- (**C,D**) and post-enhanced images (**G,H**) show stenosis in left distal ICA, proximal MCA and ACA (white arrow and arrow head). The enhancement in the bifurcation of left distal ICA is greater than that of pituitary infundibulum (curved arrow).

Two independent radiologists (M.W., M.G.; with 5 and 2 years of experience in neurovascular imaging, respectively) interpreted the vessel wall images with consensus blinded to clinical information and conventional brain images. The presence or absence of contrast enhanced lesion in the distal ICA (ophthalmic segment and communicating segment), M1 segment of the MCA and A1 segment of the ACA was determined. If the contrast enhanced lesion involved multiple arterial segments, the arterial segment with most severe stenosis will be defined as the location of the lesion. The degree of enhancement of MMV was qualitatively graded depending on its signal intensity on post-contrast images compared to corresponding pre-contrast images on Philips MR workstation (Extended Workstation, EWS)^24^: grade 0, no enhancement (Fig. 1); grade 1, mild enhancement, the signal intensity of vessel wall is less than that of the pituitary infundibulum (Fig. 2); and grade 2, strong enhancement, the signal intensity of contrast enhanced vessel wall is similar to or greater than that of the infundibulum (Fig. 3). The enhancement was regarded as concentric or eccentric patterns according to circumferential involvement. The eccentric enhancement was also considered when the thickest part of the contrast enhanced lesion was more than twice as thick as the thinnest part of that lesion^11^.

The conventional brain MR images were reviewed by one radiologist (M.W.) with 5 years experience in neuroradiology blind to vessel wall images and clinical information. Acute ischemic infarction was defined if the brain images showed hyper-intense on DWI images. Hemorrhage was not a factor in grouping patients because it was considered to be associated with a thinned media of MMV and microaneurysm^4^. The acute ischemic infarction was considered when the hyperintense was found on DWI and the time interval from onset of symptom to MR scan was less than 12 weeks^24^.

### Statistical Analysis

The categorical variables were presented as frequencies. Chi-square test was used to compare the clinical information between patients with and without acute infarction. Bonferroni correction had to be done to the results of multiple comparison. Chi-square test was also conducted to compare the patterns of concentric and eccentric enhancement. Kruskal-Wallis test was used to compare the location of moyamoya-affected vessel wall enhancement. Mann-Whitney test was utilized to compare the characteristics of intracranial vessel wall enhancement between patients with and without acute infarction. Logistic regression analysis was performed to calculate the odds ratio and corresponding 95% confidence interval of moyamoya-affected vessel wall enhancement in discriminating presence of acute ischemic infarction. Inter- and intra-reader agreement for grading the intracranial vessel wall enhancement was assessed using Cohen’s Kappa analysis before reader consensus to settle disagreements. A k value of less than 0.40 was considered poor agreement; a k value of 0.4–0.75 was characterized as fair to good agreement; a k value of greater than 0.75 indicated excellent agreement^24^. A *p* value less than 0.05 was considered statistically significant. All statistical analyses were conducted by SPSS 16.0 software (SPSS, Chicago, IL, USA).

## Additional Information

**How to cite this article:** Wang, M. *et al*. The Contrast Enhancement of Intracranial Arterial Wall on High-resolution MRI and Its Clinical Relevance in Patients with Moyamoya Vasculopathy. *Sci. Rep.* **7**, 44264; doi: 10.1038/srep44264 (2017).

**Publisher's note:** Springer Nature remains neutral with regard to jurisdictional claims in published maps and institutional affiliations.

## Figures and Tables

**Table 1:** Clinical characteristics of study population.

	MMV patients, Mean ± SD or n (%)		Chi-square test value	*P* Value
	with acute infarction (n = 25)	without acute infarction (n = 22)		
Gender, male	11 (44.0%)	9 (40.9%)	0.046	0.831
Age, years	49.1 ± 11	48.3 ± 10	—	0.805
Vascular risk factors				
Current smoker	8 (32.0%)	7 (31.8%)	0	0.989^*^
Diabetes mellitus	6 (24.0%)	3 (13.6%)	0.812	0.368^*^
Hypertension	14 (56.0%)	6 (27.3%)	3.951	0.047^*^
Hyperlipidemia	8 (32.0%)	9 (40.9%)	0.402	0.526^*^

MMV indicates moyamoya vasculopathy.

P*: The significant level is 0.05/4 according to Bonferroni correction.

**Table 2:** Characteristics of intracranial vessel wall enhancement.

	Grade 2	Grade 1	Grade 0	*p* value
Number				
MMV patients with acute infarction	26/34, 76.5%	11/22, 50%	92/190, 48.4%	0.011
MMV patients without acute infarction	8/34, 23.5%	11/22, 50%	98/190, 51.6%	
Location				
ICA	14/34, 41.2%	13/22, 59.1%	55/190, 28.9%	0.020
MCA	12/34, 35.3%	6/22, 27.3%	64/190, 33.7%	
ACA	8/34, 23.5%	3/22, 13.6%	71/190, 37.4%	
Enhancement pattern				
Concentric enhancement	22/34, 64.7%	18/22, 81.8%	—	< 0.001
Eccentric enhancement	12/34, 35.3%	4/22, 18.2%	—	

ICA, internal carotid artery; MCA, middle cerebral artery; ACA, anterior cerebral artery.
